# Six Feet under Microbiota: Microbiologic Contamination and Toxicity Profile in Three Urban Cemeteries from Lisbon, Portugal

**DOI:** 10.3390/toxins14050348

**Published:** 2022-05-16

**Authors:** Carla Viegas, Renata Cervantes, Marta Dias, Bianca Gomes, Pedro Pena, Elisabete Carolino, Magdalena Twarużek, Robert Kosicki, Ewelina Soszczyńska, Susana Viegas, Liliana Aranha Caetano

**Affiliations:** 1 H&TRC—Health & Technology Research Center, ESTeSL—Escola Superior de Tecnologia e Saúde, Instituto Politécnico de Lisboa, 1990-096 Lisbon, Portugal; r.w.cervantes@gmail.com (R.C.); martasfd@gmail.com (M.D.); bianca.gomes@estesl.ipl.pt (B.G.); pedro_migpena@hotmail.com (P.P.); etcarolino@estesl.ipl.pt (E.C.); susana.viegas@ensp.unl.pt (S.V.); liliana.caetano@estesl.ipl.pt (L.A.C.); 2 NOVA National School of Public Health, Public Health Research Centre, Universidade Nova de Lisboa, 1099-085 Lisbon, Portugal; 3 Comprehensive Health Research Center (CHRC), NOVA Medical School, Universidade NOVA de Lisboa, 1169-056 Lisbon, Portugal; 4 Department of Physiology and Toxicology, Institute of Experimental Biology, Faculty of Natural Sciences, Kazimierz Wielki University, Chodkiewicza 30, 85–064 Bydgoszcz, Poland; twarmag@ukw.edu.pl (M.T.); robkos@ukw.edu.pl (R.K.); eweso@ukw.edu.pl (E.S.); 5 Research Institute for Medicines (iMed.ULisboa), Faculty of Pharmacy, University of Lisbon, 1649-003 Lisbon, Portugal

**Keywords:** cemeteries, occupational health, *Aspergillus*, SARS-CoV-2, azole resistance, mycotoxins, cytotoxicity

## Abstract

Cemeteries are potential environmental reservoirs of pathogenic microorganisms from organic matter decomposition. This study aimed to characterize the microbial contamination in three cemeteries, and more specifically in grave diggers’ facilities. One active sampling method (impingement method) and several passive sampling methods (swabs, settled dust, settled dust filters and electrostatic dust cloths—EDC) were employed. The molecular detection of *Aspergillus* sections and SARS-CoV-2, as well as mycotoxin analysis, screening of azole resistance, and cytotoxicity measurement were also conducted. Total bacteria contamination was 80 CFU·m^−2^ in settled dust samples, reached 849 CFU·m^−2^ in EDC and 20,000 CFU·m^−2^ in swabs, and ranged from 5000 to 10,000 CFU·m^−2^ in filters. Gram-negative bacteria (VRBA) were only observed in in settled dust samples (2.00 × 10^5^ CFU·m^−2^). Regarding *Aspergillus* sp., the highest counts were obtained in DG18 (18.38%) and it was not observed in azole-supplemented SDA media. SARS-CoV-2 and the targeted *Aspergillus* sections were not detected. Mycophenolic acid was detected in one settled dust sample. Cytotoxic effects were observed for 94.4% filters and 5.6% EDC in A549 lung epithelial cells, and for 50.0% filters and 5.6% EDC in HepG2 cells. Future studies are needed in this occupational setting to implement more focused risk management measures.

## 1. Introduction

In Portugal, cemeteries are perceived as historical and religious monuments where people usually go to remember their lost loved ones [1]. The burial of corps and human remains in cemeteries facilitates the decomposition of the corpse without posing a danger to public health. Nevertheless, the World Health Organization has reported the potential impacts of cemeteries on the surrounding environment and on human health [2], focusing on soil decomposition and groundwater contamination as a public health issue [3,4], with little to no emphasis on occupational exposure. 

Among all the European countries, employers are required to prevent and assess exposure to occupational risks [5]. However, microbiologic risks are commonly less reported and, thus, under reported [6]. In fact, biological contaminants can origin a varied range of health outcomes in humans, acting as infectious, toxic, allergenic, and/or carcinogenic agents [6,7]

With the SARS-CoV-2 pandemic, and the great number of COVID-19 cases and deaths during the first waves of the pandemic, several countries faced an inordinate stress on crematoriums and cemeteries for disposal of the dead. Consequently, an increased concern was raised regarding occupational exposure of gravediggers to virus and other microbial agents. The pandemic crisis has also highlighted other specific occupational sectors of frontline workers, beyond healthcare workers, such as firefighters and waste collection workers. These occupational settings were recently characterized in Portugal regarding the occupational exposure to microbial contaminants, including SARS-CoV-2 [8]. 

The pandemic situation has prompted countries around the globe to adopt nationwide confinements to contain the spread of the SARS-CoV-2 virus. While some countries are now starting to reopen, precautions are being taken, and vaccination rates are increasing, the presence and transmission of the virus in environments where people gather is still an issue, and the risk of viral infection remains. 

The identification of occupational settings at higher risk of exposure to multi-drug resistant microbiota will enable the adoption of adequate exposure prevention measures [9]. Regarding bacteria, recent studies suggest cemetery facilities as potential environmental reservoirs of drug-resistant pathogens, reporting high frequencies of multi-drug resistant (MDR) *Escherichia coli* isolates in water samples [10] and other antibiotic resistance profiles in soil samples [10] collected from cemeteries’ surroundings. Other studies have characterized the fungal incidence and distribution in cemeteries [11]. However, to our knowledge, the role of cemeteries as potential environmental reservoirs of azole-resistant fungi has not been studied.

The emergence of fungal resistance to medical antifungal agents (mostly azoles) and the increasing incidence of azole-resistant disease due to resistant *Aspergillus fumigatus* originating from the environment have been reported [12,13,14,15]. This opportunistic fungus is responsible for severe diseases such as invasive aspergillosis in humans, with reserved prognostic in immunocompromised individuals. The identification of hotspots for the development of antifungal resistance is, therefore, of the upmost importance to prevent the dissemination of fungal resistance and to retain the use of clinical azoles among the population [12,16,17]. Additionally, and as in other occupational settings, mycotoxins contamination it is not studied until now in this occupational environment [18], creating the need of originating data to better understand the microbiota contamination in this specific environment and to identify possible links with fungal resistance profile. 

Since the lack of data regarding cemeteries’ environment limits the implementation of suitable preventive measures for cemetery workers, in this study, we characterize the microbial contamination in three cemeteries, and more specifically in grave diggers’ facilities, in the Lisbon urban area, by air and passive sampling. The molecular detection of *Aspergillus* sections and SARS-CoV-2, as well as mycotoxin analysis, screening of azole resistance and cytotoxicity measurement were also conducted to better estimate the health risks of exposure and to identify possible relations between the risk factors.

## 2. Results

### 2.1. Viable Bacterial Contamination

Total bacteria contamination ranged from 0 to 849 CFU·m^−2^ in EDC, from 5.0 × 10^3^ to 1.0 × 10^4^ CFU·m^−2^ in filters, from 0 to 2.0 × 10^4^ CFU·m^−2^ in swabs, and in settled dust samples the count was 80 CFU·m^−2^. Gram-negative bacteria load (VRBA) varied from 0 CFU·m^−2^ in EDC samples, filter samples, and swab samples, to a count of 2.00 × 10^5^ CFU·m^−2^ in settled dust samples. The greatest median value for bacterial contamination was found in surface swab samples (1.05 × 10^1^ CFU·m^−2^) whereas Gram-negative counts (2.00 × 10^1^ CFU·m^−2^) were on settled dust samples.

### 2.2. Viable Fungal Contamination

**Figure 1:**
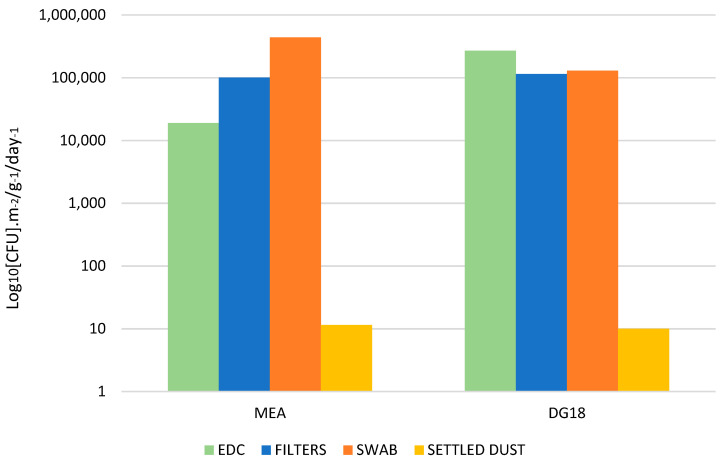
Fungal contamination distribution in the collected environmental samples (EDC CFU·m^−2^·day^−1^; Filters and Swabs CFU·m^−2^; Settled dust: CFU·g^−1^).

Fungal contamination in indoor sites was 1.00 × 10^5^ CFU·m^−2^ on MEA and 1.15 × 10^5^ CFU·m^−2^ on DG18 in filter samples; 4.40 × 10^5^ CFU·m^−2^ on MEA and 1.30 × 10^5^ CFU·m^−2^ on DG18 in floor surface swabs and 1.90 × 10^4^ CFU·m^−2^ in MEA and 2.70 × 10^5^ CFU·m^−2^ in DG18 in EDC samples. Settled dust had the highest fungal contamination, ranging from 1.15 × 10^1^ CFU·m^−2^ on MEA and 1.00 × 10^1^ CFU·m^−2^ on DG18 (Figure 1).

Concerning fungal distribution per sampling method, the highest fungal diversity was obtained through filter samples (11 species MEA; 8 species DG18), followed by EDC samples (11 species MEA; 7 species DG18) and surface swab samples (6 species MEA; 4 species DG18). The lowest fungal diversity was observed in settled dust samples (4 species MEA; 3 species DG18). *Cladosporium* sp. was the most common species obtained in filter samples (76.50% MEA; 52.38% DG18), followed by EDC samples (71.28% MEA; 39.07% DG18), settled dust samples (45.00% DG18) and surface swab samples (18.18% MEA). *Penicillium* was the prevalent genera in swab samples (63.64% MEA; 61.54% DG18), followed by EDC samples (7.38% MEA; 41.77% DG18) settled dust (26.09% MEA; 30.00% DG18) and filter samples (11.00% MEA; 16.45% DG18) (Figure 1). *Trichoderma* sp. was the most common in settled dust samples (34.78% MEA) (Figure S1—Supplementary material).

**Figure 2:**
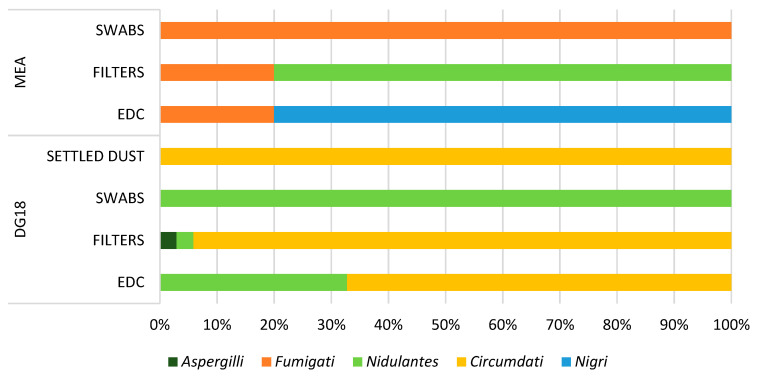
*Aspergillus* sections distribution by media and sampling method.

Regarding *Aspergillus* sp., the highest value of the genera was obtained in DG18 (18.38%) comparatively with MEA (4.11%). In DG18, the most contaminated matrixes with *Aspergillus* sp. were filter samples (27.71%). The genus was also present in surface swab samples (23.08%), EDC (11.25%), and settled dust (25.00%). On MEA, the matrices with the highest values of the genera were surface swab samples (4.55%), followed by EDC (2.79%) and filters (2.50%). The genus was not identified in settled dust samples (Figure 2).

On DG18 3 *Aspergillus* sections were identified, namely *Circumdati* (10.2%), *Nidulantes* (7.9%), and *Aspergilli* (0.2%), while on MEA 3 sections were reported, as follows: *Fumigati* (3.7%). *Nidulantes* (0.4%) and *Nigri* (0.1%). As for sections identification on EDC, 2 *Aspergillus* sections were detected on MEA (0.6% *Fumigati* and 2.2% *Nigri*) and 2 sections on DG18 (7.6% *Circumdati* and 3.7% *Nidulantes*). On filters, 2 *Aspergillus* sections were identified on MEA (0.5% *Fumigati* and 2% *Nidulantes*), while on DG18, 3 sections were identified (27.7% *Circumdati;* 0.9% *Aspergilli*; 0.9% *Nidulantes*). In swabs samples, 2 sections were identified, namely section *Fumigati* on MEA (4.6%) and section *Nidulantes* on DG18 (23.1%). In settled dust the section *Circumdati* was dominant (25%). A greater number of distinct sections was observed in samples from settled dust filters in both MEA (0.5% *Fumigati* and 2% *Nidulantes*) and DG18 (27.7% *Circumdati,* 0.9% *Nidulantes* and 0.89% *Aspergilli*) (Figure 2).

### 2.3. Azole Resistance Profile

Fungal species’ distribution in azole-supplemented media by sampling type is presented in Table 1. The most frequent fungal genus was *Cladosporium* in VCZ (1.4 × 10^5^ CFU·m^−2^·day^−1^ in EDC), SDA (1.1 × 10^5^ CFU·m^−2^ in swabs), and ITZ (7.0 × 10^4^ CFU·m^−2^·day^−1^ in EDC), followed by *C. sitophila* in SDA (1.0 × 10^5^ CFU·m^−2^ in swabs) and ITZ (6.0 × 10^4^ CFU·m^−2^·day^−1^ in EDC). Regarding the *Aspergillus* genus, it was only observed in SDA media, with the observed sections being *Nidulantes* (1.1 × 10^2^ CFU·m^−2^·day^−1^ in EDC), and *Nigri* (1.1 × 10^2^ CFU·m^−2^·day^−1^ in EDC; 1 CFU·g^−1^ in settled dust) (Table 1).

### 2.4. Detection of SARS-CoV-2 and the Targeted Fungal Sections 

Considering all the environmental samples collected, SARS-CoV-2 was not detected as well as the four *Aspergillus* sections investigated. 

### 2.5. Mycotoxins Results

From the total of 64 samples analyzed only one settled dust sample showed contamination by a single mycotoxin. The mycophenolic acid was the mycotoxin detected with a valued below the limit of quantification (20 μg/kg).

### 2.6. Cytotoxicity Evaluation

Sample dilutions from filters (2 cm^2^/mL; N = 18) and EDC (EDC average weight/20 mL; N = 18) were assessed by the MTT test for cellular metabolic activity. The obtained results are depicted in Table 2. In A549 lung epithelial cells, 17 out of 18 (94.4%) filters and 1 out of 18 (5.6%) EDC exhibited some cytotoxicity (up to 3 and 1 dilution steps, respectively). In HepG2 cells, 9 out of 18 (50.0%) filters and 1 out of 18 (5.6%) EDC were cytotoxic (up to 4 and 1 dilution steps, respectively).

### 2.7. Correlation and Comparison Analysis

From the correlation analysis (Table 3), it can be concluded that: (i) in the EDC, higher fungal counts in DG18 is related to higher counts in SDA, ITZ, and VCZ and that higher counts in SDA and ITZ are related to higher values in VCZ media; (ii) in the settled dust filters, higher fungal counts in MEA is related with higher counts in DG18, higher counts in SDA and in VCZ and that higher counts in SDA and ITZ are related to higher counts in VCZ; (iii) in swabs, higher counts in DG18 is related with counts in SDA (Table 3).

As for fungal resistance, statistically significant differences were detected in SDA (with higher values in swabs), ITZ (with higher values in EDC) and VCZ (with values in EDC) media (Table 4).

From the comparison of the fungal contamination between sampling methods, statistically significant differences were detected on MEA, and it was verified that the filters and swabs were the ones with the highest counts (Supplementary material—Table S1).

### 2.8. Correlation and Comparison Analysis 

Regarding the diversity of species found considering MEA, it was found that the settled dust (H = 1.33; D = 3.65) was the sampling method in which greater diversity was detected, as it was the one with the highest values of Shannon and Simpson indices. Of note, when the species richness (number of species) exceeds 10, Simpson’s index values are mainly influenced by the uniformity between the detected amounts of each species (Supplementary material—Table S1).

## 3. Discussion

To our knowledge this is the first attempt to assess cemeteries’ occupational environment focusing on microbial contamination and mycotoxins exposure. In addition, it was the first time that a comprehensive sampling campaign, as well as the assays employed, were used in this specific setting. The sampling approach was similar to our previous studies in different occupational environments in Portugal. The use of passive sampling methods to assess exposure, in what concerns the microbial contamination, allows to overcome the expected fluctuation due to a wide range of factors such as humidity levels, ventilation, human occupancy and their activities, environmental characteristics, water infiltrations, and outdoor air [19,20]. Additionally, in this specific setting, soil can be brought inside the workers’ facilities increasing the microbial contamination indoors [13,14,15]. The obtained results also followed the trend obtained in different studies where different sampling methods and culture media were employed. Indeed, different results were obtained in what concerns sampling methods, since swabs presented the higher bacterial contamination, while settled dust presented the highest fungal counts and species diversity. *Aspergillus* sections were more frequently observed in DG18, since this medium restricts the growth of other fungi with higher growth rates, such as Mucorales order [21,22]. *Fumigati* section was only observed in MEA corroborating also the results found in previous developed studies [23,24].

Fungal contamination, and more specifically *Aspergillus* sections distribution observed, was different in the different sampling methods as expected, since besides the media and the indoor environment assessed, also the sampling method influence the sections distribution [25]. The *Aspergillus* sections reported (*Fumigati, Circumdati, Nidulantes, Aspergilli,* and *Nigri*) have toxigenic potential [26], and some present clinical significance (*Fumigati, Nigri,* and *Aspergilli*) [23,27]. These *Aspergillus* sections are considered indicators of harmful fungal contamination [27,28], and a rigorous monitoring should be implemented to avoid their presence indoors. 

The screening of azole resistance revealed the absence of *Aspergillus* sp. in either tested azole concentration (using the EUCAST values for susceptibility testing of *Aspergillus fumigatus*). Interestingly, the presence of fungi in azole media was only confirmed by EDC sampling. The species recovered (*Cladosporium* sp. and *C. sitophila*) have no clinical relevance, and no conclusions on their antifungal resistance profile can be drawn, as the used azole breakpoint values are not defined for the species.

The correlations between sampling devices (EDC, settled dust, swabs) in different media revealed that fungal counts recovered from EDC and swabs were better correlated regarding DG18 and SDA, whereas from settled dust, MEA was best correlated with SDA. This might be related with fungal total contamination, which was higher in the azole screening for EDC (9.56 × 10^4^ CFU·m^−2^·day^−1^) and swabs (2.90 × 10^5^ CFU·m^−2^·day^−1^), compared to settled dust (5.40 × 10^1^ CFU·g^−1^·day^−1^).

The culture dependent methods allowed the observation of several *Aspergillus* sections in an extensive number of samples, with molecular tools failing the same sections detection. Despite this divergence, it is of relevance to use both assays, as they provide different information. In fact, molecular tools allow fast, specific, precise, and sensitive detection of the target microorganisms. Notably, they also can detect dormant or dead microorganisms and can differentiate toxigenic strains from regular fungal strains [24,29]. Though culture-based methods underestimate the total counts of microorganisms, these methods are vital since the microorganisms´ viability is of critical importance to predict health risks, since it affects inflammatory and cytotoxic responses [30]. This strengthens the importance of joining both culture dependent and independent methods in occupational exposure assessments [20].

As reported only one settled dust sample showed contamination by mycophenolic acid, produced mainly by *Penicillium* sp., with a value below the limit of quantification. Other occupational settings have shown higher mycotoxins contamination that might be related with many factors, such as the occupational environment characteristics (e.g., humidity, temperature, and availability of fungal nutrients) and the raw materials being used and handled [18,31]. However, this mycotoxin has the potential for causing immune dysregulation that in the long run may be related to increased oncologic morbidity and susceptibility to infections [32].

In this study, lung epithelial cells were used as a model for exposure by inhalation, and HepG2 cells as a model for hepatotoxicity [33]. Cells were incubated at controlled conditions with dilutions of wash extracts of filters and EDC. Settled dust filters revealed to be much more cytotoxic than EDC, both in A549 lung epithelial cells and hepatic cells. Although not determined statistically, this might be related with the observed differences in higher maximum total bacterial contamination in filters (1.00 × 10^4^ CFU·m^−2^) compared to EDC (8.49 × 10^2^ CFU·m^−2^). Gram-negative bacteria contamination and fungal contamination (MEA and DG18) were similar among filters and EDC, thus, probably not responsible for the observed differences in cytotoxicity. Fungal diversity, however, might explain differences in cytotoxicity. For example, *Aspergillus* sp. is highly cytotoxic [34], with differences among *Aspergillus* sections [35,36,37]. In this study, augmented *Aspergillus* sp. distribution in filters (compared to EDC) consisted of sections *Nidulantes* (only in filters, MEA), *Circumdati* (higher contamination in filters, DG18) and *Aspergilli* (only in filters, DG18). Of these, *Nidulantes* is among the most pathogenic to humans *Aspergillus* species, being mycotoxigenic (able to produce sterigmatocystin, penicillin, cotanin, and nidulotoxin) [38]. Most fungal metabolites, such as mycotoxins, are cytotoxic to different cellular structures. The best described mycotoxins produced by *Aspergillus* species, aflatoxins and ochratoxins, can act towards target cells, cellular structures, and their internal processes [39].

The IC50 levels were 0.016 g/mL in one EDC only, both in A549 cells and HepG2 cells. Regarding filters, IC50 levels ranged from 0.25 to 1 cm^2^/mL in A549 cells, and from 0.125 to 1 cm^2^/mL in HepG2 cells. Previous studies reported a cytotoxicity of *Aspergillus* metabolites in A549 cells as ranging from 44 to 61 µM [40]. Moreover, in other attributes such as particle size, MVOCs (not assessed in this study) may have cytotoxic effects [41].

## 4. Conclusions

Overall, this study provided a useful contribute to unveil the microbial contamination in cemeteries and estimate workers exposure to the microbial contamination characterized, since the sampling locations were defined based on the tasks developed by the workers and places where they spent more time. This is a very specific context with no available data published on microbiological contamination and exposure of the workers involved in the activities developed. Additionally, the data produce allowed to characterize exposure and identify some measures to prevent exposure. It was also possible to reinforce the positive features of the sampling approaches followed (combining active with the passive sampling methods), as well as the assays applied (use of different culture media). 

Future studies are needed in this occupational setting regarding mycotoxins and cytotoxicity measurement to identify potential fungal producers and or triggers for mycotoxin production allowing to implement more focused risk management measures.

## 5. Materials and Methods

### 5.1. Graveyards Assessed 

**Figure 3:**
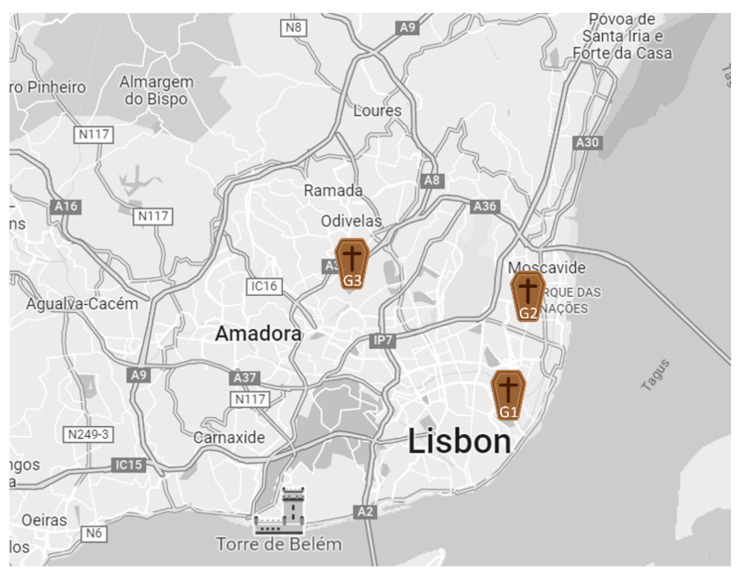
Cemetery locations in Lisbon city.

This study sampling campaign was conducted between May and June 2021 in three cemeteries located in Lisbon (Figure 3). There were 41 workers in Cemetery 1 (G1), 22 in Cemetery 2 (G2), and 13 in Cemetery 3 (G3), and all worked in regular 8-h shifts. In G1 there were 1 cemetery coordinator, 1 foreman, 7 administrative workers, 18 gravediggers, 8 gravediggers from the crematorium, 4 drivers of heavy machinery and special vehicles, 1 paver, and 1 locksmith. In G2 there were 1 cemetery coordinator, 1 foreman, 5 administrative workers, 5 gravediggers, 8 gravediggers from the crematorium, and 2 auxiliary workers. In G3 there were 1 cemetery coordinator, 1 foreman, 3 administrative workers, 5 gravediggers, 2 auxiliary workers and 1 concierge, accounting for a total of 76 workers in all the assessed cemeteries. The sampling sites were the Administrative service, Repository room, Crematory, Bar, Kitchen, Canteen (tables and Self-Service area), male and female locker room, and drivers’ and gravediggers’ locker room. 

### 5.2. Sampling Approach and Characterization through Culture Dependent-Methods

**Figure 4:**
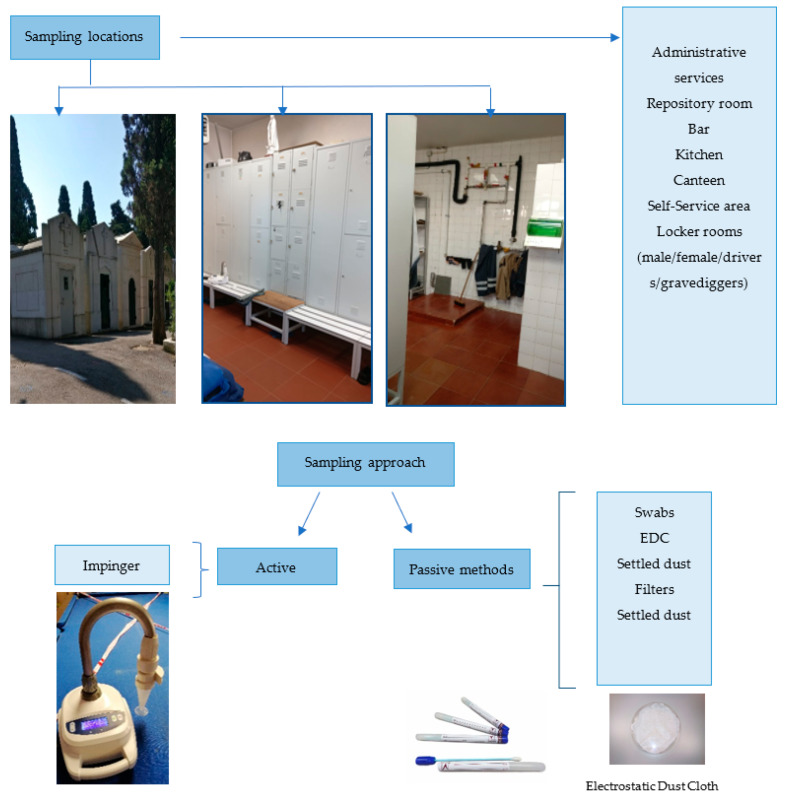
Sampling approach applied in the cemetery’s assessment.

A multi-sampling approach protocol was performed by using active and passive sampling methods and through normal working days (Figure 2). An impinger device—Coriolis μ air sampler (Bertin Technologies, Montigny-le-Bretonneux, France)—was employed for SARS-CoV-2 detection. The passive sampling methods used in the implemented sampling campaign were settled dust, surfaces swabs and electrostatic dust cloths (EDC) (Figure 4). 

Settled dust was collected through a vacuum cleaner (HOOVER Brave BV71_BV10 A2, Solon, OH, USA) with 1 × 4 collector filter attached (also used for further analyses) and a composite sample of the settled dust filters was obtained by vacuuming all the identified sampling sites [8,42]. Floor surfaces from the same sampling sites were swabbed following the same procedures already reported [21,22,23,24,25,26,27,28,29,30,31,32,33,34,35,36,37,38,39,40,41,42,43] to allow microbial quantification. The EDC were placed above 1.5 m high on a shelf in each sampling site for 30 days. All samples obtained (settled dust, floor surface swabs, filters, EDC) were preserved in sterilized bags or in transport tubes (swabs) and transported under refrigeration (0–4 °C) to the laboratory for further analyses [42].

Swabs obtained from swabbing the floor were washed with 1 mL of 0.1% Tween 80 saline (0.9% NaCl) for 30 min on the orbital shaker (250 rpm, 30 min). The same procedure was applied on a piece (2 cm^2^) of each filter used from vacuuming. A composite sample with the settled dust was washed in a ratio of 1 g per 9.1 mL of 0.1% Tween 80 saline (0.9% NaCl) for 30 min at 250 rpm [42]. EDC were weighted and processed with 20 mL of the same washing solution. 

After incubation at 27 °C for 5–7 days for fungi (MEA and DG18) and at 30 °C (TSA) and 35 °C (VRBA) for 7 days for mesophilic bacteria and coliforms (Gram negative bacteria), respectively, microbial quantification was achieved (colony-forming units, CFU·g^−1^, CFU·m^−2^, CFU·m^−2^·day^−1^) as previously published [8]. Fungal species were identified microscopically following instructions reported by De [44]. Negative controls were used to ensure the inexistence of background contamination, namely: culture media (all samples) and control samples´ extracts (vacuuming filters, swabs, EDC) without prior use were analyzed to the same assays.

### 5.3. Azole Resistance Screening

The extracts from the passive sampling methods from cemeteries were screened for azole resistance using an adaptation of the EUCAST method and breakpoints for *Aspergillus fumigatus* [45,46]. For that purpose, Sabouraud dextrose agar (SDA) (Frilabo, Maia, Portugal) was used either alone (as control) or supplemented with the following medical azoles: 4 µg/mL itraconazole (ITZ), 2 µg/mL voriconazole (VCZ), and 0.5 µg/mL posaconazole (PSZ). The washed extracts of the collected passive samples (prepared as described in Section 2.2) were inoculated in the SDA supplemented media, and the media plates were incubated at 27 °C, to allow the growth of all fungi present in sample. As negative control, the reference strain *A. fumigatus* ATCC 204305 (provided by National Health Institute Doutor Ricardo Jorge) was used, and a pan-azole-resistant *A. fumigatus* (also provided by National Health Institute Doutor Ricardo Jorge, IP) was used as positive control. After three of incubation, fungal colonies were counted and prepared for microscopically identification [47]. 

### 5.4. Sampling and Molecular Detection of SARS-CoV-2 and Targeted Aspergillus Sections

Concerning SARS-CoV-2, composite surface samples were obtained by swabbing defined areas from each sampling site in each cemetery (Administrative service, Repository room, Crematory, Bar, Kitchen, Canteen (Tables and Self-Service area), Male and female locker room, drivers’ and gravediggers’ locker room) (Figure 2), using sterile cotton swabs moistened in Buffer NVL (specific for SARS-COV-2 assessment) and following the same steps applied on swabs applied to assess other microorganisms (fungi and bacteria). 

Air samples of 600 L were obtained in all sampling locations employing the impinger Coriolis μ air sampler (Bertin Technologies, Montigny-le-Bretonneux, France) with a flow rate of 300 L/min collected into a conical vial containing 5 mL Buffer NVL (NZY Viral RNA Isolation kit (MB40701) component). 

Samples were kept refrigerated (until 4 °C) for a maximum of 24 h before RNA was extracted from the obtained sample (1.5 mL in surface samples and 5 mL in air samples) with the NZY Viral RNA Isolation kit, from Nzytech, following to manufacturer’s instructions. One step-RT qPCR was performed using NZYSpeedy One-step RT-qPCR probe Master Mix and specific procedures were followed (Supplementary Material) with primers and probes published by CDC (available on: https://www.cdc.gov/coronavirus/2019-ncov/lab/rt-pcr-panel-primer-probes.html, accessed on 23 September 2020), (Supplementary material—Table S2). qPCR was performed on BioRad CFX96 PCR machine. To detect possible PCR inhibitors an internal control was added to each PCR. 

Samples extracts (8.8 mL) from passive sampling (excluding surface swabs) were used for molecular detection of *Aspergillus* sections (*Fumigati, Circumdati, Flavi* and *Nidulantes*) following the steps as previous published [42] (Supplementary material—Table S3). 

### 5.5. Mycotoxins Analysis

Thirty-nine samples were screened for mycotoxins presence: in 18 samples collected from the filters, in 18 EDC and in 3 settled dust samples as previous reported [8]. Thirty-eight mycotoxins were analyzed by HPL-MS (HPLC) Nexera (Shimadzu, Tokyo, Japan) with a mass spectrometry detector API 4000 (Sciex, Foster City, CA, USA). The Limits of Detection (LOD) obtained for each mycotoxin with the analytical method used are presented in Table S4 (Supplementary Materials).

### 5.6. Cytotoxicity Analyses

The cytotoxic effect of filter and EDC samples was evaluated on samples’ extracts using the 3-(4,5-dimethylthiazol-2-yl)-2,5-diphenyltetrazolium bromide (MTT) assay at 510 nm, as previously described [48]. The cells used to assess the cytotoxicity were the human lung epithelial (A549) and the HepG2 cells. Briefly, cells were maintained in Eagle’s Minimum Essential Medium (MEM) supplemented with 10,000 units penicillin and 10 mg/mL streptomycin in 0.9% NaCl and foetal bovine serum (Sigma-Aldrich, Burlington, MA, USA), and 0.25% (*w*/*v*) Trypsin 0.53 mM EDTA was used for cell detachment. After cell counting (Scepter™ 2.0 Cell Counter, Merck, Kenilworth, NJ, USA), 100 µL cell suspension (densities of 2.5 × 10^5^ cells/mL) was transferred to a 96-well plate and exposed to diluted samples for 48 h at 5% CO_2_, 37 °C, and humid atmosphere. The cytotoxicity was measured (ELISA LEDETECT 96, biomed Dr. Wieser GmbH; MikroWin 2013SC software), and the lowest sample concentration dropping absorption to <50% of cell metabolic activity (IC50) was considered the threshold toxicity level.

### 5.7. Statistical Analyses

Data obtained were analyzed using SPSS statistical software, version 26.0 for Windows. The results were considered significant at the 5% significance level. To test the normality of the data, the Shapiro–Wilk test was used. To study the relationship between fungal counts (MEA and DG18) and fungal counts on azole resistance screening (SDA ITZ, VCZ, and PSZ) in each sampling method, Spearman correlation coefficient was applied, since the assumption of normality was not observed. To compare the fungal contamination and azole resistance screening between the sampling methods, the Kruskal–Wallis test was applied, since the normality assumption was not observed. To assess species diversity, Simpson and Shannon indices, given by $Shannon\;Index\;\left(H\right)=-\overset{s}{\underset{i=1}{\sum }}p_{i}ln\left(p_{i}\right)$ and $Simpson\;Index\;\left(D\right)=\frac{1}{\sum_{i=1}^{s}p_{i}^{2}}$, were used, where *p_i_* is the proportion (n_i_/n) of individuals of one particular species found (n_i_) divided by the total number of individuals found (n).

## Figures and Tables

**Table 1:** Fungal species’ distribution in azole-supplemented media by type of environmental samples.

		SDA		ITZ		VCZ		PSZ	
Matrice	Species	CFU·m^−2^·Day^−1^/g^−1^/m^2^	%	CFU·m^−2^·Day^−1^/g^−1^/m^−2^	%	CFU·m^−2^·Day^−1^/g^−1^/m^−2^	%	CFU·m^−2^·Day^−1^/g^−1^/m^−2^	%
EDC	*A.* section *Nidulantes*	1.06 × 10^2^	0.1	0.0	0.0	0.0	0.0	0.0	0.0
	*A.* section *Nigri*	1.06 × 10^2^	0.1	0.0	0.0	0.0	0.0	0.0	0.0
	*Cladosporium* sp.	5.20 × 10^4^	54.4	7.01 × 10^4^	36.9	1.46 × 10^5^	64.1	1.00 × 10^4^	16.5
	*Chrysosporium* sp.	0.0	0.0	0.0	0.0	2.00 × 10^4^	8.8	0.0	0.0
	*C. sitophila*	2.06 × 10^4^	21.6	6.01 × 10^4^	31.6	5.03 × 10^4^	22.0	5.03 × 10^4^	83.3
	*Penicillium* sp.	2.17 × 10^4^	22.7	6.00 × 10^4^	31.5	0.0	0.0	1.06 × 10^4^	0.2
	Other species	1.06 × 10^3^	1.1	0.0	0.0	1.16 × 10^4^	5.1	0.0	0.0
	Total	9.56 × 10^4^	100.0	1.90 × 10^5^	100.0	2.28 × 10^5^	100.0	6.04 × 10^4^	100.0
FILTERS	*Cladosporium* sp.	1.05 × 10^4^	33.9	5.00 × 10^2^	33.3	1.00 × 10^4^	50.0	0.0	0.0
	*C. sitophila*	1.00 × 10^3^	3.2	5.00 × 10^2^	33.3	5.00 × 10^2^	25.0	6.00 × 10^3^	92.3
	*Fusarium verticilloides*	0.0	0.0	5.00 × 10^2^	33.3	0.0	0.0	0.0	0.0
	*Penicillium* sp.	1.90 × 10^4^	61.3	0.0	0.0	5.00 × 10^2^	25.0	5.00 × 10^2^	7.7
	Other species	5.00 × 10^2^	1.6	0.0	0.0	0.0	0.0	0.0	0.0
	Total	3.10 × 10^4^	100.0	1.50 × 10^3^	100.0	2.00 × 10^3^	100.0	6.50 × 10^3^	100.0
SWABS	*Cladosporium* sp.	1.10 × 10^5^	37.9	0.0	0.0	4.00 × 10^4^	57.1	0.0	0.0
	*C. sitophila*	1.00 × 10^5^	34.5	3.00 × 10^4^	60.0	0.0	0.0	3.00 × 10^4^	75.0
	*Penicillium* sp.	5.00 × 10^4^	17.2	1.00 × 10^4^	20.0	2.00 × 10^4^	28.6	0.0	0.0
	*Rhizopus* sp.	0.0	0.0	0.0	0.0	1.00 × 10^4^	14.3	0.0	0.0
	*Trichoderma* sp.	0.0	0.0	1.00 × 10^4^	20.0	0.0	0.0	1.00 × 10^4^	25.0
	Other species	3.00 × 10^4^	10.3	0.0	0.0	0.0	0.0	0.0	0.0
	Total	2.90 × 10^5^	100.0	5.00 × 10^4^	100.0	7.00 × 10^4^	100.0	4.00 × 10^4^	100.0
SETTLED DUST	*Aureobasidium* sp.	4.00	7.4	0.0	0.0	0.0	0.0	0.0	0.0
	*A. section Nigri*	1.00	1.9	0.0	0.0	0.0	0.0	0.0	0.0
	*Cladosporium* sp.	0.0	0.0	4.00	57.1	1.06 × 10^2^	92.2	0.0	0.0
	*Chrysosporium* sp.	0.0	0.0	0.0	0.0	2.00	1.7	0.0	0.0
	*C. sitophila*	1.50	2.8	0.0	0.0	0.0	0.0	0.0	0.0
	*Geotrichum* sp.	0.0	0.0	0.0	0.0	0.0	0.0	1.00	25.0
	*Penicillium* sp.	4.45 × 10^1^	82.4	3.00	42.9	6.00	5.2	3.00	75.0
	Other species	3.00	5.6	0.0	0.0	1.00	0.9	0.0	0.0
	Total	5.40 × 10^1^	100.0	7.00	100.0	1.15 × 10^2^	100.0	4.00	100.0

**Table 2:** Distribution of IC50 values in filter and EDC from cemeteries.

Dilution Step	Filters			EDC		
	IC50	A549	HepG2	IC50	A549	HepG2
1	1	13	6	0.016	1	1
2	0.5	3	1	0.008	0	0
3	0.25	1	0	0.004	0	0
4	0.125	0	2	0.002	0	0
(-)		1	9		17	17

(-) no cytotoxicity.

**Table 3:** Heatmap of the study of the relationship between fungal contamination (MEA and DG18) and fungal resistance (SDA ITZ, VCZ, and PSZ) in each sampling method. Results of the Spearman correlation coefficient.

Method		Media	Fungi (CFU·m^−2^/m^−^^2^·Day^−^^1^)	Fungal Resistance (CFU·m^−^^2^/m^−2^·Day^−^^1^)			
			DG18	SDA	ITZ	VCZ	PSZ
EDC	Fungi (CFU·m^−2^·day^−1^)	MEA	0.242	0.105	0.284	0.340	−0.035
		DG18		0.606 **	0.510 *	0.692 **	0.345
	Fungal resistance (CFU·m^−2^·day^−1^)	SDA			0.446	0.514 *	−0.034
		ITZ				0.628 **	0.261
		VCZ					0.411
Filters	Fungi (CFU·m^−2^)	MEA	0.598 **	0.507 *	0.188	0.675 **	0.162
		DG18		0.271	0.132	0.460	−0.257
	Fungal resistance (CFU·m^−2^)	SDA			0.378	0.623 **	0.238
		ITZ				0.478 *	0.452
		VCZ					0.225
Swabs	Fungi (CFU·m^−2^)	MEA	0.221	0.105	−0.387	−0.166	−0.183
		DG18		0.646 **	0.228	0.405	0.257
	Fungal resistance (CFU·m^−2^)	SDA			0.283	0.074	0.045
		ITZ				0.214	0.037
		VCZ					0.399

*. Correlation is significant at the 0.05 level (2-tailed). **. Correlation is significant at the 0.01 level (2-tailed). The colour indicates the correlation level found.

**Table 4:** Comparison of fungal contamination (on MEA and DG18) and fungal resistance (on SDA, ITZ, VCZ and PSZ) between sampling methods. Results of the Kruskal–Wallis test.

		Sampling Method	Ranks		Test Statistics			Kruskal–Wallis Multiple Comparisons Test
			n	Mean Rank	Kruskal–Wallis H	df	p	
Fungi (CFU·m^−2^/m^−2^·day^−1^)	MEA	EDC	18	19.58	6.943	2	0.031 *	EDC ≠ Filter (*p* = 0.048)
		Filter	18	32.17				
		Swabs	18	30.75				
		Total	54					
	DG18	EDC	18	31.42	4.948	2	0.084	
		Filter	18	30.17				
		Swabs	18	20.92				
		Total	54					
Fungal resistance (CFU·m^−2^/m^−2^·day^−1^)	SDA	EDC	18	24.67	16.003	2	0.000 *	EDC ≠ Swabs (*p* = 0.017)
		Filter	18	18.81				Filter ≠ Swabs (*p* = 0.000)
		Swabs	18	39.03				
		Total	54					
	ITZ	EDC	18	36.58	12.915	2	0.002 *	EDC ≠ Filter (*p* = 0.002)
		Filter	18	20.92				EDC ≠ Swabs (*p* = 0.031)
		Swabs	18	25.00				
		Total	54					
	VCZ	EDC	18	36.94	12.729	2	0.002 *	EDC ≠ Filter (*p* = 0.002)
		Filter	18	20.78				EDC ≠ Swabs (*p* = 0.030)
		Swabs	18	24.78				
		Total	54					
	PSZ	EDC	18	30.17	5.591	2	0.061	
		Filter	18	31.33				
		Swabs	18	21.00				
		Total	54					

* Statistically significant differences at the 5% significance level.

## Supplementary Materials

The following supporting information can be downloaded at: https://www.mdpi.com/article/10.3390/toxins14050348/s1, Figure S1—The prevalent fungal genera on MEA and DG18 in samples from different matrices; Table S1—Assessment of species diversity (Shannon’s and Simpson’s indices) in each sampling method; Table S2—Novel Coronavirus (2019-nCoV) Real-time RT-PCR Panel Primers and Probes.; Table S3— Sequence of primers and TaqMan probes used for real-time PCR; Table S4—LOD values for the samples analysed.
