# Supplementary material for: Six Feet under Microbiota: Microbiologic Contamination and Toxicity Profile in Three Urban Cemeteries from Lisbon, Portugal
Source: Toxins (Basel). 2022 May 16;14(5):348. doi: 10.3390/toxins14050348 (PMC9146229; doi:10.3390/toxins14050348)
Supplement: Supplementary file 1 [file toxins-14-00348-s001.zip › toxins-1698399-supplementary.pdf]

Supplementary Materials: Six Feet under Microbiota: Microbiologic Contamination and Toxicity Profile in Three Urban Cemeteries from Lisbon, Portugal

Carla Viegas, Renata Cervantes, Marta Dias, Bianca Gomes, Pedro Pena, Elisabete Carolino, Magdalena Twarużek, Robert Kosicki, Ewelina Soszczyńska, Susana Viegas and Liliana Aranha Caetano

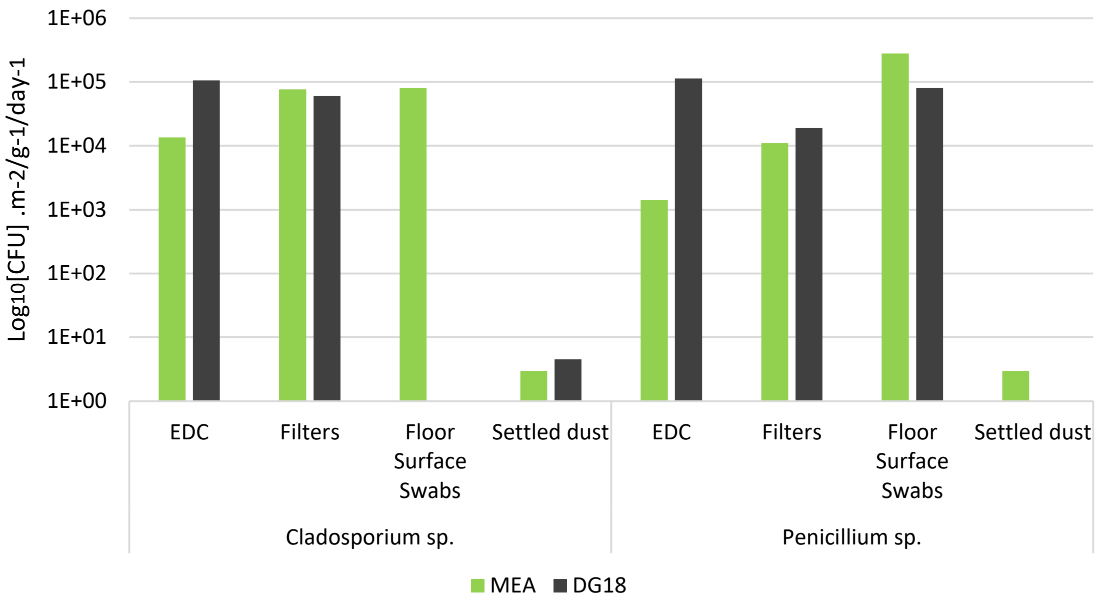

Figure S1. The prevalent fungal genera on MEA and DG18 in samples from different matrices (EDC: CFU.m^{-2}.day^{-1}; Fillters and Swabs CFU.m $^{-2}$; Settled dust: CFU.g $^{-1}$).

Table S1. Assessment of species diversity (Shannon's and Simpson's indices) in each sampling method.

**Method**	**Fungi**	**Culture media**	**Diversity indices**	
		**MEA (CFU. m^{-2}.day^{-1}/ m^{-2}/g^{-1})**	**Shannon Index (H)**	**Simpson Index (D)**
**EDC**	*Alternaria* sp.	530.786	1.03	1.86
	Aureobasidium sp.	110.157		
	C.sitophila	114.157		
	Chrysosporium sp.	13571.639		
	F. verticilloides	2441.614		
	A. section *Fumigati*	106.157		
	A. section *Nigri*	424.628		
	Penicillium sp.	1404.042		
	Trichoderma sp.	106.157		
	Ulocladium sp.	116.157		

FILTER	*Allternaria* sp.	500	0.95	1.67
	Aureobasidium sp.	500		
	C.sitophila	4000		
	Chrysonilia sp.	500		
	Chrysosporium sp.	1500		
	Cladosporium sp.	76500		
	A. section *Fumigati*	500		
	Fusarium equisetti	1000		
	A. section Nidulantes	2000		
	Penicillium sp.	11000		
	Ulocladium sp.	2000		
Settled Dust	*Aureobasidium* sp.	1.5	1.33	3.65
	Cladosporium sp.	3		
	Penicillium sp.	3		
	Trichoderma sp.	4		
SWAB	*Aureobasidium* sp.	10000	1.13	2.23
	C.sitophila	40000		
	Chrysosporium sp.	10000		
	Cladosporium sp.	80000		
	A. section *Fumigati*	20000		
	Penicillium sp.	280000		

Samples were kept refrigerated (-until 4 °C) for a maximum of 24 h before RNA was extracted from the isolated sample (5 mL in air samples and 1.5 ml in surface samples) with the NZY Viral RNA Isolation kit, from Nzytech, according to manufacturer's instructions. One step-RT qPCR was performed using NZYSpeedy One-step RT-qPCR probe Master Mix with primers and probes published by CDC (available on https://www.cdc.gov/coronavirus/2019-ncov/lab/rt-pcr-panel-primer-probes.html), which detect two viral gene regions (N1 and N2). qPCR was performed on BioRad CFX96 PCR machine. In each analysis a positive (a SARS-CoV-2 positive sample) and a negative (water) sample was included. Furthermore, in order to detect possible PCR inhibitors an internal control was added to each PCR.

Table S2. Novel Coronavirus (2019-nCoV) Real-time RT-PCR Panel Primers and Probes.

Name	Description	Oligonucleotide Sequence (5′>3′)	Label1
2019-nCoV_N1-F	2019-nCoV_N1 Forward Primer	5′-GAC CCC AAA ATC AGC GAA AT-3′	None
2019-nCoV_N1-R	2019-nCoV_N1 Reverse Primer	5′-TCT GGT TAC TGC CAG TTG AAT CTG-3′	None
2019-nCoV_N1-P	2019-nCoV_N1 Probe	5′-FAM-ACC CCG CAT TAC GTT TGG TGG ACC-BHQ1-3′	FAM, BHQ-1
2019-nCoV_N2-F	2019-nCoV_N2 Forward Primer	5′-TTA CAA ACA TTG GCC GCA AA-3′	None
2019-nCoV_N2-R	2019-nCoV_N2 Reverse Primer	5′-GCG CGA CAT TCC GAA GAA-3′	None

2019-nCoV_N2-P	2019-nCoV_N2 Probe	5′-FAM-ACA ATT TGC CCC CAG CGC TTC AG-BHQ1-3′	FAM, BHQ-1
Spike In		TATAA Universal RNA Spike I	FAM

Fungal DNA was extracted using the ZR Fungal/Bacterial DNA MiniPrep Kit (Zymo Research, Irvine, USA) and molecular identification was performed by Real Time PCR (qPCR) using the CFX-Connect PCR System (Bio-Rad). Reactions included 1× iQ Supermix (Bio-Rad, Portugal), 0.5 μM of each primer, and 0.375 μM of TaqMan probe in a total volume of 20 μl. Amplification followed a three-step PCR: 40 cycles with denaturation at 95 °C for 30 s, annealing at 52 °C for 30 s, and extension at 72 °C for 30 s (Supplementary material – Table S3). A non-template control and a positive control consisting of DNA obtained from a reference that belonged to the culture collection of the Reference Unit for Parasitic and Fungal Infections, Department of Infectious Diseases of the National Institute of Health, from Dr. Ricardo Jorge. These strains have been sequenced for ITS, B-tubulin, and Calmodulin.

Table S3. Sequence of primers and TaqMan probes used for real-time PCR.

**Fungal Species/Sections Targeted**	**Sequences**	**Reference**
	Flavi	
Forward Primer	5′–GTCCAAGCAACAGGCCAAGT–3′	Mayer, Z.; Bagnara, A.; Färber, P.; Geisen, R. Quantification of the copy number of nor-1, a gene of the aflatoxin biosynthetic pathway by real-time PCR, and its correlation to the cfu of Aspergillus flavus in foods. Int. J. Food Microbiol. 2003, 82, 143–151.
Reverse Primer	5′–TCGTGCATGTTGGTGATGGT–3′	
Probe	5′–TGTCTTGATCGGCGCCCG–3′	
	Fumigati	
Forward Primer	5′–CGCGTCCGGTCCTCG–3′	Cruz-Perez, P.; Buttner, M.P.; Stetzenbach, L.D. Detection and quantitation of Aspergillus fumigatus in pure culture using polymerase chain reaction. Mol. Cell. Probes. 2001, 15, 81–88.
Reverse Primer	5′–TTAGAAAAATAAAGTTGGGTGTCGG–3′	
Probe	5′–TGTCACCTGCTCTGTAGGCCCG–3′	
	Circumdati	
Forward Primer	5′–CGGGTCTAATGCAGCTCCAA–3′	[12]
Reverse Primer	5′–CGGGCACCAATCCTTTCA–3′	
Probe	5′–CGTCAATAAGCGCTTTT–3′	
	Nidulantes	
Forward Primer	5′–CGGCGGGGAGCCCT–3′	United States Environmental Protection Agency (EPA), About the National Exposure Research Laboratory (NERL), (n.d.). Available online: http://www.epa.gov/nerlcwww/moldtech.html
Reverse Primer	5′–CCATTGTTGAAAGTTTTGACTGATCTTA–3′	
Probe	5′–AGACTGCATCACTCTCAGGCATGAAGTTCAG–3′	

In all samples, 38 mycotoxins were analyzed by HPL-MS ((HPLC) Nexera (Shimadzu, Tokyo, Japan) with a mass spectrometry detector API 4000 (Sciex, Foster City, CA, USA). The mycotoxin concentration was calculated using external calibration. The Limits of Detection (LOD) obtained for each mycotoxin with the analytical method used are presented in Table S4.

Table S4. LOD values for the samples analysed.

Type of Sample	Mycotoxins	LOD (μg/kg)
EDC	15-Acetyldeoxynivalenol	8
	3-Acetyldeoxynivalenol Aflatoxin B_1	4
	Aflatoxin B_2	1
	Aflatoxin G_1	1
	Aflatoxin G_2	1
	Aflatoxin M_1	1
	Deepoxydeoxynivalenol	5
	Deoxynivalenol	8
	Deoxynivalenol-3-glucoside	5
	Diacetoxyscirpenol	2
	Fumonisin B_1	4
	Fumonisin B_2	3
	Fusarenon X	10
	Griseofulvin	2
	HT-2 toxin	4
	Mevinolin	7
	Monoacetoxyscirpenol	2
	Mycophenolic acid	3
	Neosolaniol	3
	Nivalenol	4
	Ochratoxin A	2
	Ochratoxin B	2
	Patulin	8
	Roquefortine C	2
	Sterigmatocystin	1
	T-2 tetraol	2
	T-2 toxin	2
	T-2 triol	5
	Zearalanone	2
	Zearalenone	1
	α-Zearalanol	2
	α-Zearalenol	2
	β-Zearalanol	2
	β-Zearalenol	3
Filters	15Acetyldeoxynivalenol	8
	3-Acetyldeoxynivalenol Aflatoxin B_1	4
	Aflatoxin B_2	1
	Aflatoxin G_1	1
	Aflatoxin G_2	1

	Aflatoxin M_1	1
	Deepoxydeoxynivalenol	5
	Deoxynivalenol	8
	Deoxynivalenol-3-glucoside	5
	Diacetoxyscirpenol	2
	Fumonisin B_1	4
	Fumonisin B_2	3
	Fusarenon X	10
	Griseofulvin	2
	HT-2 toxin	4
	Mevinolin	7
	Monoacetoxyscirpenol	2
	Mycophenolic acid	3
	Neosolaniol	3
	Nivalenol	4
	Ochratoxin A	2
	Ochratoxin B	2
	Patulin	8
	Roquefortine C	2
	Sterigmatocystin	1
	T-2 tetraol	2
	T-2 toxin	2
	T-2 triol	5
	Zearalanone	2
	Zearalenone	1
	α-Zearalanol	2
	α-Zearalenol	1
	β-Zearalanol	3
	β-Zearalenol	2
Settled dust	15Acetyldeoxynivalenol	20
	3-Acetyldeoxynivalenol Aflatoxin B_1	10
	Aflatoxin B_2	2
	Aflatoxin G_1	1
	Aflatoxin G_2	2
	Aflatoxin M_1	1
	Deepoxydeoxynivalenol	2
	Deoxynivalenol	11
	Deoxynivalenol-3-glucoside	8
	Diacetoxyscirpenol	2
	Fumonisin B_1	1
	Fumonisin B_2	2
	Fusarenon X	12
	Griseofulvin	5
	HT-2 toxin	3
	Mevinolin	11
	Monoacetoxyscirpenol	4
	Mycophenolic acid	6
	Neosolaniol	5
	Nivalenol	7

Ochratoxin A	3
Ochratoxin B	4
Patulin	18
Roquefortine C	5
Sterigmatocystin	2
T-2 tetraol	5
T-2 toxin	3
T-2 triol	10
Zearalanone	2
Zearalenone	2
α-Zearalanol	4
α-Zearalenol	3
β-Zearalanol	4
β-Zearalenol	3
